# Evolution of Mutation Rate in Astronomically Large Phytoplankton Populations

**DOI:** 10.1093/gbe/evaa131

**Published:** 2020-07-09

**Authors:** Marc Krasovec, Rosalind E M Rickaby, Dmitry A Filatov

**Affiliations:** 1 Department of Plant Sciences, University of Oxford, United Kingdom; 2 Department of Earth Sciences, University of Oxford, United Kingdom

**Keywords:** mutation rate, phytoplankton evolution, mutation accumulation, *Emiliania huxleyi*, Lewontin’s paradox, effective population size, codon bias

## Abstract

Genetic diversity is expected to be proportional to population size, yet, there is a well-known, but unexplained lack of genetic diversity in large populations—the “Lewontin’s paradox.” Larger populations are expected to evolve lower mutation rates, which may help to explain this paradox. Here, we test this conjecture by measuring the spontaneous mutation rate in a ubiquitous unicellular marine phytoplankton species *Emiliania huxleyi* (Haptophyta) that has modest genetic diversity despite an astronomically large population size. Genome sequencing of *E. huxleyi* mutation accumulation lines revealed 455 mutations, with an unusual GC-biased mutation spectrum. This yielded an estimate of the per site mutation rate *µ* = 5.55×10^−10^ (CI 95%: 5.05×10^−10^ – 6.09×10^−10^), which corresponds to an effective population size *N*_e _∼ 2.7×10^6^. Such a modest *N*_e_ is surprising for a ubiquitous and abundant species that accounts for up to 10% of global primary productivity in the oceans. Our results indicate that even exceptionally large populations do not evolve mutation rates lower than ∼10^−10^ per nucleotide per cell division. Consequently, the extreme disparity between modest genetic diversity and astronomically large population size in the plankton species cannot be explained by an unusually low mutation rate.

**Significance**

Surprisingly little is known about evolutionary genetic processes in astronomically large populations of marine phytoplankton. This study reports the first estimate of a key evolutionary parameter—spontaneous mutation rate—for a major domain of life, the Haptophytes. It also helps to shed light on the Lewontin’s paradox—disproportionately modest genetic diversity in large populations—the disparity that is particularly pronounced in large plankton populations.

## Introduction

The level of genetic diversity in a population is determined by the balance between the new mutations occurring in the population and the loss of polymorphisms by stochastic processes (drift) and selection (Leffler et al. 2012; Ellegren and Galtier 2016). More mutations are expected to occur in a larger population because there are more individuals to mutate. In addition, drift is weaker in larger populations (Crow and Kimura 1970), thus larger populations are expected to contain more genetic diversity. On the other hand, selection is expected to be more powerful in populations of larger size, potentially allowing selection to reduce mutation rate to lower values in larger populations (Lynch 2010; Sung, Ackerman, et al. 2012; Lynch et al. 2016), which may explain the well-known phenomenon of relatively low genetic diversity in large populations (Lewontin 1974; Leffler et al. 2012; Corbett-Detig et al. 2015; Ellegren and Galtier 2016; Filatov 2019; Xu et al. 2019). Here, we test this idea by measuring the spontaneous mutation rate in a unicellular eukaryotic marine coccolithophore *Emiliania huxleyi* (Haptophyta) that has an astronomically large population size and thus would be expected to evolve to a very low mutation rate if this rate is determined by efficacy of selection.

*Emiliania huxleyi* is ubiquitous and so abundant in modern oceans that its seasonal blooms are visible from space. Like most other coccolithophores, it produces coccoliths (calcite shields with species-specific shapes) at the cell surface. Due to its abundance, *E. huxleyi* is thought to be the main calcite producer on Earth (Paasche 2001; Daniels et al. 2014, 2016; Rembauville et al. 2016) affecting the global CO_2_ budget and carbon cycle. Due to its ecological importance and ease of culturing, *E. huxleyi* became a model phytoplankton species with a significant body of on-going work devoted to the interplay between coccolithophore abundance, climate change and the global carbon cycle (Rickaby et al. 2007; Iglesias-Rodriguez et al. 2008). However, surprisingly little is known about evolutionary genetic processes in populations of marine phytoplankton generally (Rengefors et al. 2017) and *E. huxleyi* populations in particular (Bendif et al. 2019; Filatov 2019).

Based on the large population sizes of marine plankton species, their genetic diversity is often assumed to be very high (Read et al. 2013). Yet, recent studies of genetic diversity in a number of marine phytoplankton species revealed a surprisingly low level of single nucleotide polymorphism (Blanc-Mathieu et al. 2017; Filatov 2019; Rastogi et al. 2020). In particular, genetic diversity in a world-wide sample of *E. huxleyi* is only π∼0.006 per silent site across the genome (Filatov 2019)—similar to the level of polymorphism in *Arabidopsis thaliana* (1001 Genomes Consortium 2016), twice lower than in marine unicellular green algae *Ostreococcus tauri* (Blanc-Mathieu et al. 2017) and at least three times lower than in *Drosophila melanogaster* (Langley et al. 2012). Patterns of genetic diversity (e.g., very low linkage disequilibrium) in the *E. huxleyi* genome rule out any trivial explanations for this low diversity, such as high clonality or recent expansion from a very small population (Filatov 2019). However, an unusually low mutation rate may account, at least partly, for this low genetic diversity (Xu et al. 2019). For example, the mutation rate in another group, ciliates, was reported to be two to three orders of magnitude lower than in other studied eukaryotes (Sung, Tucker, et al. 2012; Long et al. 2018). No estimates of mutation rates are available for any Haptophyte species, making it difficult to assess *E. huxleyi* mutation rate even to an order of magnitude. To address this, we measured the spontaneous mutation rate in the diploid *E. huxleyi* strain RCC1242 (=CCMP1516) for which a ∼167-Mb long-genome sequence was published previously (Read et al. 2013).

## Materials and Methods

### Mutation Accumulation Experiment

We performed a mutation accumulation (MA) experiment with the diploid *E. huxleyi* strain RCC1242 (=CCMP1516). We followed the protocol previously developed by Krasovec (Krasovec et al. 2016) for MA experiments in a liquid medium. The MA experiment included 15 MA lines and lasted 8 months. The size of the MA experiment was planned, assuming the mutation rate is of the order of 10^−10^ per nucleotide per cell division, as found in a few other phytoplankton species (Ness et al. 2012; Krasovec et al. 2017, 2019), but with the possibility to expand the size of the experiment should the *E. huxleyi* mutation rate prove to be much lower. The initial line was obtained from a single cell by dilution and used to inoculate 15 MA lines kept in 24-well plates at 20 °C in F2 medium. Serial bottlenecks every 14 days were used to reduce the efficiency of selection by decreasing the population size of the MA lines. At each bottleneck, the cell culture was counted with a Beckman Multisizer Coulter Counter to calculate the number of cell divisions in the time interval and inoculate the MA lines in fresh media with one cell by dilution following a previously developed protocol (Krasovec et al. 2016, 2017; Krasovec, Sanchez-Brosseau, et al. 2018). The average cell division per day of the MA lines between each bottleneck could be used as a proxy of the fitness throughout the experiment. We used a linear correlation to test a change in fitness over the time of the experiment with R v3.5.1.

### Genome Resequencing and Identification of De Novo Mutations

DNA of the 15 MA lines and the initial culture were extracted with the DNeasy Plant Mini Kit of QIAGEN following the standard instructions. Genomic libraries were prepared and sequenced at the Wellcome Trust Centre for Human Genetics (WTCHG) at the University of Oxford, UK. Genomic DNA was quantified using Qubit (Invitrogen) and the size profile analyzed using eGel (Thermo Fisher, 1% EX Agoarose). Input material was normalized to 300 ng prior to fragmentation and library preparation. Fragmentation was performed by mechanical shearing to an average size of 300 bp (Covaris S2 series; duty Cycle—10%, intensity—5.00, cycles/Bursts—200, time—60 s). Automated library preparation was performed using the Apollo 324 prep system (Wafergen, PrepX ILMN 32i, 96 sample kit) and standard Illumina multiplexing adapters following manufacturer’s protocol up to pre-PCR amplification. Libraries were PCR amplified (10 cycles) on a Tetrad (Bio-Rad) using the NEBNext High-Fidelity 2× PCR Master Mix (NEB) and in-house unique dual indexing primers (based on Lamble et al. [2013]). Post-PCR purification performed using Agencourt Ampure XP (Beckman Coulter; ratio 1:1) before combining. Individual libraries were normalized using Qubit, and the size profile was analyzed on the 2200 or 4200 TapeStation (Agilent). Individual libraries were normalized and pooled together accordingly. The pooled library was diluted to ∼10 nM for storage. The 10-nM library was denatured and further diluted prior to loading on the sequencer. Paired-end sequencing was performed using a HiSeq4000 150-bp platform (Illumina, HiSeq 3000/4000 PE Cluster Kit, and 300 cycle SBS Kit), generating a raw read count of >34 million reads per sample (table 1).

**Table 1:** Nuclear De Novo Mutations Identified in the 15 *Emiliania huxleyi* MA Lines

Lines	NCBI Sample ID	Gb	Cov	G*	Callable Sites	Gen	*N*_bs_
Eh_mut_A	SAMN13932576	6.80	65	70.9	118,881,543	210	34
Eh_mut_E	SAMN13932577	6.08	58	71.0	119,008,226	209	22
Eh_mut_G	SAMN13932578	6.79	48	71.7	120,234,404	209	40
Eh_mut_H	SAMN13932579	7.35	70	70.0	117,447,180	221	44
Eh_mut_J	SAMN13932583	8.19	32	70.2	117,688,670	267	40
Eh_mut_M	SAMN13932584	7.00	40	71.7	120,186,108	285	19
Eh_mut_N	SAMN13932585	6.40	33	70.2	117,776,564	273	30
Eh_mut_O	SAMN13932586	7.32	31	69.8	116,961,626	291	26
Eh_mut_R	SAMN13932587	6.68	51	71.6	120,132,427	202	21
Eh_mut_S	SAMN13932588	7.51	59	69.5	116,500,955	214	52
Eh_mut_T	SAMN13932589	7.61	54	71.1	119,224,148	209	47
Eh_mut_U	SAMN13932590	7.77	59	69.9	117,252,421	200	20
Eh_mut_X	SAMN13932580	6.76	29	62.3	104,532,863	232	23
Eh_mut_Y	SAMN13932581	6.40	47	71.7	120,278,083	240	25
Eh_mut_Z	SAMN13932582	7.74	61	71.3	119,602,275	218	12
Eh_T0	SAMN13932591	6.87	32	73.1	122,565,059	—	—

Note.—Sequence data are available from NCBI (bioproject PRJNA532543). Gb, the amount of sequence data generated (Gigabase); Cov, the average sequence coverage; G*, the % of callable genome; Gen, the number of MA generations; *N*_bs_, the number of de novo nucleotide substitutions per line.

PCR duplicates were removed with GATK v3.4-46 (McKenna et al. 2010) and reads were mapped against the reference genome of the RCC1242 strain (NCBI accession: GCA_000372725.1) (Read et al. 2013) with BWA mem v.0.7.12 (Li and Durbin 2009). For organelles, we used the reference mitochondrion NC_005332.1 (Sánchez Puerta et al. 2004) and the reference chloroplast NC_007288.1 (Sánchez Puerta et al. 2005) genomes. To calculate the mutation rate per haploid organelle genome, the average ploidy levels of the organelle genomes per cell (estimated from read coverage for organellar relative to nuclear genomes) were taken into account.

The BAM files were sorted with Samtools v.1.2 (Li et al. 2009) and variants called with HaplotypeCaller from GATK v3.4-46 (McKenna et al. 2010) following the best practice recommendations (RealignerTargetCreator and IndelRealigner). To avoid false positives during de novo mutation identification, we apply several criteria used in previous studies with PCR confirmation of mutation candidates (Keightley et al. 2015; Krasovec, Chester, et al. 2018). Criteria were: *1*) callable sites were defined with a threshold of 20 mapping quality and 2) a minimal coverage of 20 in the MA line and ancestral genomes; 3) sites covered by >150× were removed to exclude repetitive regions; 4) the alternative allele was supported with a minimal coverage of 1/3rd of the total coverage; 5) candidates within an insertion–deletion were removed with Bcftools v1.2 (options SnpGap = 5); and 6) all de novo mutations were manually checked in the mpileup files generated by Samtools v.1.2 for all MA lines and the ancestral genome. To test the reliability of in silico identification of de novo mutations, we randomly selected de novo mutation candidates for manual verification based on PCR and Sanger sequencing. All manually checked mutations were confirmed to be true positives (supplementary table S3, Supplementary Material online). The effect of de novo mutations was determined with snpEff v4.3 (Cingolani et al. 2012). To detect any bias in the mutation distribution, we compared the mutation distribution using χ^2^ and binomial tests against the null hypothesis assuming that mutations appear independently and randomly in the genome.

### Mutation Bias and GC Content Evolution

To detect a potential mutation bias in the mutation spectrum, we calculated *R1* and *R2* mutation rates for GC to AT and AT to GC nucleotide mutations, respectively. With the numbers of mutations from GC to AT and AT to GC and the number of sites in the genome *GCn* and *ATn*, *R1* = (GC to AT) / *GCn* and *R2* = (AT to GC) / *ATn*. Equilibrium GC content (*GC*_eq_), was calculated as *GC*_eq_=*R2* / (*R1*+*R2*). The equilibrium GC content is the GC content reached by the mutation process alone, that is, the GC content, where the numbers of mutations from GC to AT and AT to GC are equal.

### Codon Bias

Codon bias in *E. huxleyi* was measured with the effective number of codons (ENCs; Wright 1990) as implemented in software CodonW (http://codonw.sourceforge.net). First, we ran a correspondence analysis to generate the hilo.coa files containing the preferred and unpreferred codons (based on a two-way χ^2^ contingency test) and the fop.coa files. Then, we calculated the frequency of optimal codons and the ENCs gene by gene using the files generated by the previous correspondence analysis. Last, we calculated the strength of selected codon usage bias (*S*) from the codon bias in the highly expressed *E. huxleyi* genes using the method of Sharp et al. (2005), but using the mutation spectrum observed in our MA experiment, as we did previously in the analysis of codon bias in *Phaeodactylum tricornutum* (Krasovec and Filatov 2019). *S* was calculated for the entire genome, as well as for 500 most actively expressed genes. Expression data for this analysis were obtained from a previous study (Huff and Zilberman 2014) (raw data SRR847300 from the bioproject PRJNA201680). Raw reads were aligned against the transcriptome with RSEM v.1.2.31 (Li and Dewey 2011) to obtain the fpkm values for each gene. *S* was calculated only for amino acids encoded by two codons where one is the preferred one (Phe, Tyr, His, Gln, Asn, Lys, Asp, and Glu). Furthermore, the fpkm values were used to test the effect of expression level on the mutation rate by comparing expression at the genes with and without a mutated site.

## Results

### Mutation Accumulation Experiment and *E. huxleyi* Mutation Rate

To measure the spontaneous mutation rate in *E. huxleyi*, we conducted a MA experiment (Halligan and Keightley 2009) that included 15 MA lines grown under standard lab conditions for 232 generations on an average, totaling 3,480 generations across all MA lines. To exclude selection and allow all mutations, including deleterious ones, to be fixed, the effective population size of MA lines was reduced by serial bottlenecking—reduction of the population to one cell every 2 weeks. From the cell counts at each bottleneck time, we estimated 1.17 generations per day on an average (supplementary table S1, Supplementary Material online) and an average effective population size of *N*_e _= 7.6 (estimated from the harmonic mean of cell number) throughout the experiment. The generation time average did not change over the time of the experiment (Spearman correlation test, *P* value = 0.8355).

**Fig. 1.**
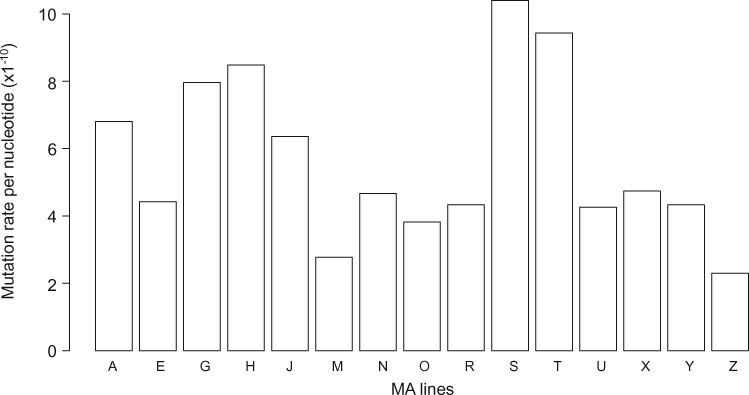
Nuclear mutation rate per MA line. Average number of mutations per MA line is ∼30.33 (SD = 11.84). The number of mutations per MA line differs from the theoretical distribution assuming equal mutation rate across the lines (Pearson’s χ^2^ test, χ^2^ = 37.733, *P* value = 0.0006).

In order to identify the mutations accumulated during the MA experiment, we used Illumina high-throughput sequencing to sequence the genomes of the MA lines at the beginning and the end of MA experiment (table 1). The analysis of nuclear genome sequence data from MA lines identified 455 de novo single nucleotide mutations (tables 1 and 2 and supplementary table S2, Supplementary Material online). All mutations that we verified manually with PCR and Sanger sequencing, were confirmed to be true positives (supplementary table S3, Supplementary Material online). With 117,713,833 callable sites per MA line on an average (table 1), the spontaneous mutation rate in the nuclear genome is *µ* = 5.55×10^−10^ (Poisson CI 95%: 5.05×10^−10^ – 6.09×10^−10^) per nucleotide per cell division. The mutation rate variation between the lines was significant (fig. 1, Pearson’s χ^2^ test, *P* value = 0.0006), such as observed, for example, in *Chlamydomonas reinhardtii* and *Caenorhabditis elegans* (Ness et al. 2015; Konrad et al. 2018). The per haploid genome per cell division mutation rate (*U* = genome size in bp×µ) in *E. huxleyi* is *U *=* *167×10^6^×5.55×10^−10^ = 0.092, whereas the mutation rate per coding sequence (39,635,709 nt of annotated CDS) (Read et al. 2013) is *U*_cds _= 0.022. Based on µ and synonymous intraspecific polymorphism from 17 *E. huxleyi* strains (Filatov 2019) (*π*_s_ ∼0.006), the estimate of effective population size in *E. huxleyi* is *N*_e_ = *π*_s _/ (4×*µ*) ∼2.7 million. In addition, we identified seven and two de novo mutations in the mitochondrial and the chloroplast genomes, respectively (supplementary table S2, Supplementary Material online). The resulting estimates of per site per cell division mutation rates are *µ*_mt _*=* 1.45×10^−9^ (Poisson CI 95%: 5.82×10^−10^ – 2.98× 10^−9^) and *µ*_cl _= 1.76×10^−10^ (Poisson CI 95%: 2.13×10^−11^ – 6.43×10^−10^) for mitochondria and chloroplasts, respectively.

**Table 2:** Nuclear De Novo Mutations (*Nmut*) Affecting Different Types of Functional Regions

Mutation Effect	*Nmut*
UTR	15
Intergenic	329
Intron	7
Missense_variant	67
Splice_region	3
Start_lost	1
Stop_gained	1
Synonymous_variant	32

Note.—The average number of callable sites per MA line was 30,362,830 for coding sequences and 87,351,003 for noncoding sequences.

### Distribution of De Novo Mutations in the Genome

We detected de novo mutations in all three genomic compartments—nuclear, mitochondrial, and chloroplast DNA, with a significantly higher mutation rate in mitochondria (*µ*_mt _*=* 1.45×10^−9^) compared with the nuclear genome (2×2 contingency χ^2^ = 5.44, *P* value = 0.0196). Although the chloroplast mutation rate (*µ*_cl _= 1.76×10^−10^) appears to be lower than the nuclear rate (*µ* = 5.55×10^−10^), this difference is not significant (2×2 contingency χ^2^ = 2.28, *P* value = 0.1309). Too few chloroplast and mitochondrial mutations were detected to analyze the distributions within these genomes.

Most of the nuclear de novo mutations occurred in noncoding regions (354), whereas 32 and 69 mutations occurred at synonymous and nonsynonymous positions, respectively, in annotated coding regions. These numbers do not differ significantly from the expectations based on the proportions of callable noncoding, synonymous, and nonsynonymous positions in the *E. huxleyi* genome (Binomial test, ns). Furthermore, we did not detect any significant correlations of local mutation rate with genomic features. Finally, gene expression was not significantly different between genes with and without a mutated site (fpkm average of genes with a mutated site = 34.69, fpkm average of genes without a mutated site = 21.62, Student’s test, *P* value = 0.6628).

DNA methylation (and associated deamination of methyl-cytosines) is a major contributor of mutations in the eukaryotic genomes (Holliday and Grigg 1993). If methylation contributes to the rate and pattern of mutations in *E. huxleyi*, we would expect to detect a lack of CpG dinucleotides in the genome (e.g., as is the case in mammals) and an excess of de novo C to T transitions at such dinucleotides. CpG represents 10.8% of all dinucleotides in the genome of *E. huxleyi*, which does not deviate from what is expected given the genomic GC-content (χ^2^ test, ns). Furthermore, the total number of de novo mutations from CpG to CpH is 10.3%, that is, there is no excess of mutations at CpG sites (χ^2^ test, ns). These results indicate that the effect of CpG methylation on mutation rate is low or absent in *E. huxleyi.*

**Fig. 2.**
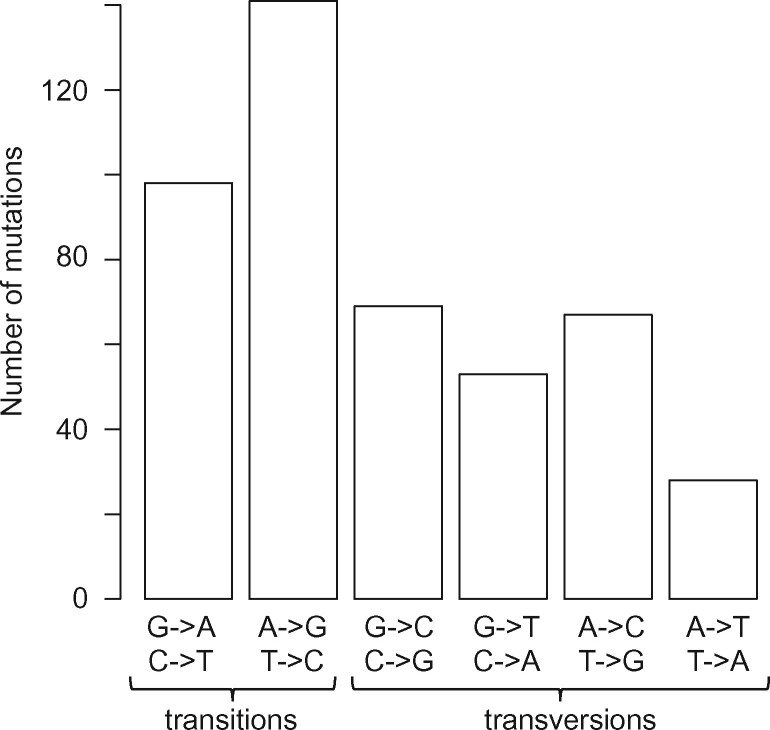
Mutation spectrum of *Emiliania huxleyi*. Transitions occur more frequently than transversions, with observed transition/transversion ratio *k *=* *1.11.

The analysis of mutational patterns (fig. 2) revealed significantly more AT to GC mutations compared with GC to AT mutations (207 vs. 151, respectively, binomial test, *P* value = 0.0036 with probability 50:50). This indicates that the *E. huxleyi* mutation spectrum has a significant GC-bias, which makes *E. huxleyi* the first eukaryotic species with a GC-biased mutation spectrum detected in a direct MA experiment, though, indirect inference of the mutation spectrum from sequence polymorphism and divergence indicated that the genome of plant *Coffea canephora* may also have a slightly GC-biased mutation spectrum (Clement et al. 2017). With an average of *µ*_GC->AT_*=*0.381×*µ*_AT->GC_, the equilibrium GC-content of the *E. huxleyi* genome is *GC*_eq_ = 72.42%. The actual GC-content is slightly lower than the *GC*_eq_ for the total genome (65.7%) and the noncoding regions (63.0%). In the coding sequence, the actual GC is 69.1%, close to *GC*_eq_ due to the very high GC-content at third-codon positions (GC3s = 84.2%).

### Codon Bias and Long-Term Effective Population Size

High GC-content at third-codon positions is due to strong codon usage bias in *E. huxleyi*. The preferred codons (listed in supplementary table S4, Supplementary Material online) almost always end with G or C and a widely used measure of codon bias – ENC (Wright 1990), averaged across all genes was ENC = 38.17, whereas for 500 strongest and weakest expressed genes ENC is 35.52 and 39.03, respectively. Using the method of Sharp et al. (2005) we estimated the strength of selected codon usage bias (*S*) for amino acids encoded by one preferred and one unpreferred codons (Phe, Tyr, His, Gln, Asn, Lys, Asp, and Glu; see supplementary table S4, Supplementary Material online). For 500 most actively expressed genes, where selection for codon usage is expected to be the strongest, the frequency of optimal codons was 0.8859, corresponding to *S *=* *1.084, which is similar to the strength of selected codon bias in other organisms with large populations, such as *Drosophila*, nematodes, and bacteria (Sharp et al. 2010). This allows us to estimate long-term effective population size, given that evolution of codon bias is a slow process (Lawrence and Ochman 1997) that “averages” over short-term changes in population size and efficacy of selection (Sharp et al. 2010). Assuming selective advantage (*s*) of the preferred compared with unpreferred codons to be of the order of 10^−6^–10^−7^, as suggested by analyses of codon bias in *Drosophila* (Akashi 1997; Powell and Moriyama 1997) and bacteria (Sharp et al. 2010), the long-term effective population size in *E. huxleyi* and its ancestral species is of the order *N*_e_ = *S* / 4*s* ∼ 1.084 / 4×10^−6^ ∼271,000 to *N*_e_ ∼ 1.084 / 4×10^−7^ ∼2,710,000. The latter estimate is almost identical to our genetic diversity-based estimate *N*_e_ = *π*_s _/ (4×*µ*) ∼2.7 million. Given that this estimate is independent of the mutation rate and genetic diversity values, the correspondence between the genetic diversity-based and codon bias-based estimates of *N*_e_ is reassuring.

## Discussion

### *Emiliania huxleyi* Mutation Rate

Here, we reported the first estimate of spontaneous mutation rate for a Haptophyte species. *Emiliania huxleyi* per-nucleotide per cell division mutation rate (*µ* = 5.55×10^−10^) is close to estimates for other eukaryotic plankton, such as the diatom *P. tricornutum* (Krasovec et al. 2019) (*µ* = 4.77×10^−10^) or unicellular green algal species *O. tauri* (Krasovec et al. 2017) (*µ* = 4.79×10^−10^) and *Chlamydomonas reinhardtii* (Ness et al. 2012) (*µ* = 3.23×10^−10^). This similarity of mutation rates across Haptophytes, Stramenopiles, and Chlorophyta suggests that the mutation rate of the order *µ* ∼ 5×10^−10^ is typical for unicellular eukaryotes regardless of their phylogenetic affinities. Ciliates represent a notable exception to this, with *Paramecium tetraurelia* having an order of magnitude lower mutation rate (Sung, Tucker, et al. 2012), possibly due to their peculiar life cycle and the presence of two genomes in macro- and micronuclei (Long et al. 2018).

Our estimates of mutation rates in the nuclear, mitochondrial, and chloroplast genomes of *E. huxleyi* reveal that, similar to animals (Denver et al. 2000; Xu et al. 2012; Konrad et al. 2017) and diatoms (Krasovec et al. 2019), but contrary to plants (Drouin et al. 2008; Ossowski et al. 2010), haptophytes have a higher mitochondrial than nuclear mutation rate. The chloroplast mutation rate in the diatoms (Krasovec et al. 2019) and green plants (Smith 2015; Smith and Keeling 2015) is lower than that of the nuclear genome. The estimates of nuclear (*µ* = 5.55×10^−10^) and chloroplast (*µ*_cl _= 1.76×10^−10^) mutation rates in *E. huxleyi* show a difference in the same direction, although the difference between the two rates is not significant.

### Is the Lab-Based Mutation Rate Estimate Representative of That in the Open Ocean?

The spontaneous mutation rate may be affected by environmental conditions (Jiang et al. 2014; Liu and Zhang 2019). It is not possible to accurately reconstruct all the diversity of environmental conditions for a species that is ubiquitous in the world oceans and inhabits environments ranging from the tropics to the Arctic. However, it is possible to use the rich fossil record for this species (Raffi et al. 2006) to calibrate the rate of the molecular clock and compare it with the mutation rate found in the lab. Based on the fossil record, *E. huxleyi* evolved from the genus *Gephyrocapsa* ∼290 ka (Raffi et al. 2006). This is consistent with the conclusions of an integrated analysis of fossil and genome sequence data from *E. huxleyi* and four *Gephyrocapsa* species (Bendif et al. 2019), which demonstrated that 290 kyr corresponds to the divergence between *E. huxleyi* and *Gephyrocapsa muellerae*. Given sequence divergence *d*_s_ ∼ 3% (Bendif et al. 2019) and the time of divergence (T∼290 kyr) between these species, the mutation rate in the open ocean can be estimated as *µ*_year_ = *d*_s _/ 2T ≈ 5.18×10^−8^ per year. Given the maximal rate of cell division achieved under optimal lab conditions in our experiment (∼1.17 per day), *E. huxleyi* in the wild should have <300 generations per year, providing the minimal estimate *µ*_gen_ = 5.18×10^−8^/ 300 = 1.73×10^−10^ for the per generation mutation rate in the wild. Using our MA-based estimate of *µ*_gen_, we can infer the number of generations per year in the wild to be ∼93 (= 3.45×10^−8 ^/ 5.55×10^−10^) or one generation in ∼4 days. This estimate appears realistic because environmental conditions in nature are not always optimal for growth and *E. huxleyi* blooms require specific conditions (Tyrrell and Merico 2004). This suggests that the lab-based estimate of mutation rate (*µ*_gen_ = 5.55×10^−10^) accurately reflects *E. huxleyi* mutation rate in the wild.

### Effective Population Size and Evolution of Mutation Rates

**Fig. 3.**
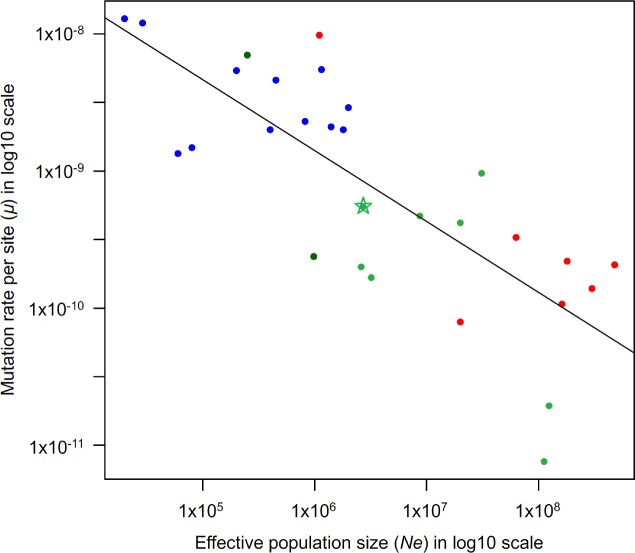
Effective population sizes (*N*_e_) and per site mutation rates (µ) in *Emiliania huxleyi* (star) and other species (blue, animals; dark green, plants; light green, unicellular eukaryotes; red, bacteria) There is a strong negative correlation between *N*_e_ and *µ* (Pearson corr. test, *ρ* = −0.78 and *P* value = 5.706×10^−7^). Data used for this plot are listed in supplementary table S5, Supplementary Material online.

Mutation rates vary widely among organisms and their evolution is thought to be driven by natural selection (Lynch 2010; Sung, Ackerman, et al. 2012), which is reflected by the negative correlation between per-nucleotide mutation rates and effective population size (fig. 3). As most mutations are deleterious (Muller 1950), in most circumstances selection is expected to favor a reduction in the overall mutation rate (Lynch 2010). However, the strength of such selection is thought to be relatively weak (Drake et al. 1998) and unable to overcome genetic drift in small populations. Such weak selection to reduce mutation rate can be effective only in populations of large size, where drift is weak (Sung, Ackerman, et al. 2012). Here, we used a unicellular eukaryotic phytoplankton species with astronomically large population size to test whether selection in such a large population is able to push its mutation rate below the usual 10^−9 ^– 10^−10^ range typical for unicellular eukaryotic species.

Our analysis revealed that despite the astronomically large *E. huxleyi* populations, selection is unable to reduce the mutation rate <10^−10^ per nucleotide per cell division. One possibility to explain this apparent minimum to the mutation rate is that ∼10^−10^ represents the limit to how low the mutation rate can be reduced due to intrinsic biochemical constraints of replication and error correction cellular machinery (Kunkel and Erie 2015). Very few known species, regardless of their biology and ecology, can reach a per site mutation rate <10^−10^. The only known organisms with <10^−10^ mutation rate are ciliates (Sung, Tucker, et al. 2012; Long et al. 2018). Another possibility is that despite the astronomical census population size of *E. huxleyi*, its effective population size (*N*_e_) is relatively modest, that is, drift is relatively high due to demography or other reasons, as reflected by the modest genetic diversity of this species (Filatov 2019). Indeed, the estimates of *E. huxleyi N*_e_ from its genetic diversity (*N*_e_ = *π*_s _/ (4×*µ*) ∼2.7 million) and codon bias *N*_e_ ∼ *S*/4*s* = 1.084 / 4×10^−7^ ∼2.7 million are both smaller than the estimates of *N*_e_ in other marine phytoplankton species, including the green algae *O. tauri* (*N*_e_ ∼ 12 million; Blanc-Mathieu et al. 2017) and the diatom *P. tricornutum* (*N*_e_ ∼ 8.7 million; Krasovec et al. 2019).

The reasons for extreme disparity between the astronomically large census population size and modest effective population size in *E. huxleyi* remain uncertain, though, it is clear that given very low linkage disequilibrium, this disparity is unlikely to be caused by periodic asexual reproduction of this species (Filatov 2019). Neither population size changes (Filatov 2019) nor speciation bottlenecks can explain the limited genetic diversity of *E. huxleyi* because the population size of this abundant species remained large even during its recent (∼290 ka) speciation from *Gephyrocapsa* (figure 2c in Bendif et al. [2019]). Linked selection (“genetic draft”) may potentially account for modest *N*_e_ and limited genetic diversity, though, the amount of selection needed for this appears very high (figure 7 in Filatov [2019]). Our study rules out the possibility that the extreme disparity between modest genetic diversity and astronomically large population size in the plankton species is due to an unusually low mutation rate that is expected to evolve in very large populations (fig. 3 and Sung, Ackerman, et al. 2012). The population genetic processes dominating huge populations of marine plankton species, such as *E. huxleyi* studied here, remain poorly understood (Rengefors et al. 2017) and deserve more attention from the research community.

## Supplementary Material

evaa131_Supplementary_Data

## Notes

**Data deposition:** Newly generated raw read sequence data have been deposited at SRA database (NCBI) under the bio project PRJNA532543.
