# Supplementary material for: Evolution of Mutation Rate in Astronomically Large Phytoplankton Populations
Source: Genome Biol Evol. 2020 Jul 9;12(7):1051–9. doi: 10.1093/gbe/evaa131 (PMC7486954; doi:10.1093/gbe/evaa131)
Supplement: evaa131_Supplementary_Data [file evaa131_supplementary_data.pdf]

Table S1. Average of number of generations per day between bottleneck times.

	Day of the MA experiment							
**Line**	**14**	**28**	**42**	**56**	**70**	**84**	**98**	**112**
**J**	0.901	1.195	1.441	1.218	1.516	1.311	1.289	1.289
**O**	0.964	1.256	1.514	1.549	1.305	1.184	1.354	1.354
**M**	0.957	1.238	1.492	1.581	1.445	1.216	1.193	1.193
**N**	0.857	1.003	1.396	1.498	1.359	1.049	1.225	1.225
**Y**	1.179	0.911	1.000	1.102	0.919	1.067	1.331	1.469
**X**	1.128	1.033	1.076	1.459	0.801	1.048	1.339	1.468
**Z**	1.092	0.953	0.943	1.281	0.722	0.991	1.222	1.294
**A**	1.110	0.862	0.986	1.118	1.398	1.410	1.207	1.096
**E**	0.918	0.761	0.849	1.119	1.417	1.500	1.239	1.263
**G**	1.012	0.866	0.940	1.125	1.413	1.501	1.244	1.177
**H**	1.075	0.967	1.035	1.152	1.460	1.566	1.379	1.368
**R**	0.895	1.146	1.427	1.475	1.255	0.972	0.999	0.999
**S**	0.910	1.206	1.440	1.502	1.418	1.199	1.265	1.265
**T**	0.876	1.130	1.433	1.461	1.408	1.109	1.177	1.177
**U**	0.839	1.160	1.499	1.495	1.209	0.927	0.993	0.993

Table S1 continued

	Day of the MA experiment							
**Line**	**126**	**140**	**154**	**168**	**182**	**196**	**210**	**224**
**J**	1.016	0.992	1.156	0.988	1.113	1.291	1.263	1.117
**O**	1.354	1.115	1.277	1.184	1.360	1.502	1.358	1.175
**M**	1.119	1.120	1.284	1.174	1.348	1.429	1.357	1.203
**N**	1.225	0.954	1.101	1.118	1.293	1.512	1.428	1.259
**Y**	1.241	1.040	1.077	1.160	0.989	0.999	1.06639	
**X**	1.198	1.040	1.123	1.321	1.043	1.020	1.04308	
**Z**	1.170	0.916	0.983	1.025	0.937	0.993	1.02279	
**A**	1.291	1.281	1.093	1.021	1.158			
**E**	1.246	1.336	1.061	1.035	1.204			
**G**	1.267	1.287	1.020	1.009	1.072			
**H**	1.305	1.337	1.070	1.094	1.057			
**R**	1.002	1.130	1.108	1.030	1.018			
**S**	1.000	1.245	0.998	0.795	1.056			
**T**	1.050	1.202	1.026	0.802	1.072			
**U**	0.993	1.123	1.087	0.973	1.010			

Table S2. Base substitution mutations.

MA line	Contig	Pos	Ref	Alt
**A**	NW_005200000.1	6425	T	C
**A**	NW_005196346.1	17626	G	A
**A**	NW_005200087.1	82538	G	A
**A**	NW_005200461.1	115836	A	G
**A**	NW_005200169.1	133663	G	A
**A**	NW_005200000.1	146973	C	T
**A**	NW_005196094.1	174103	C	A
**A**	NW_005201318.1	245096	T	A
**A**	NW_005201231.1	247500	G	C
**A**	NW_005201165.1	300281	A	C
**A**	NW_005195584.1	355947	T	G
**A**	NW_005200011.1	476872	G	A
**A**	NW_005201851.1	479118	T	G
**A**	NW_005198167.1	542632	T	C
**A**	NW_005200881.1	703403	A	G
**A**	NW_005199684.1	736412	T	G
**A**	NW_005200011.1	848908	A	C
**A**	NW_005201287.1	855000	C	G
**A**	NW_005198244.1	925441	C	G
**A**	NW_005196756.1	1071410	G	C
**A**	NW_005201287.1	1135139	A	G
**A**	NW_005201403.1	88478	A	T
**A**	NW_005201478.1	9503	C	A
**A**	NW_005200272.1	89902	C	G
**A**	NW_005200853.1	124572	C	T
**A**	NW_005201732.1	283687	C	T
**A**	NW_005198965.1	606136	C	T
**A**	NW_005199442.1	61643	G	A
**A**	NW_005200218.1	96220	G	A
**A**	NW_005200784.1	864777	G	C
**A**	NW_005198017.1	469614	G	T
**A**	NW_005196664.1	18761	T	A
**A**	NW_005198900.1	735135	T	C
**A**	NW_005196681.1	146039	T	G
**E**	NW_005195988.1	8754	T	G
**E**	NW_005199219.1	13808	C	A
**E**	NW_005196020.1	27697	C	G
**E**	NW_005196756.1	33470	C	G
**E**	NW_005200188.1	45237	A	C
**E**	NW_005197576.1	82859	A	C
**E**	NW_005200249.1	84797	G	C
**E**	NW_005200000.1	117296	A	C
**E**	NW_005196681.1	136448	T	G
**E**	NW_005201392.1	157462	T	G
**E**	NW_005198467.1	205557	G	C
**E**	NW_005201349.1	228077	G	A
**E**	NW_005199770.1	254827	C	G
**E**	NW_005196379.1	398268	A	G
**E**	NW_005196756.1	430704	A	C

Table S2 continued

MA line	Contig	Pos	Ref	Alt
**O**	NW_005198544.1	8715	T	C
**O**	NW_005197506.1	21158	A	G
**O**	NW_005200543.1	46907	G	C
**O**	NW_005198161.1	47835	G	T
**O**	NW_005200272.1	75300	T	C
**O**	NW_005200941.1	99457	G	T
**O**	NW_005200951.1	169168	A	G
**O**	NW_005201912.1	197780	C	T
**O**	NW_005197576.1	225871	T	C
**O**	NW_005200250.1	227655	T	C
**O**	NW_005196379.1	250047	T	C
**O**	NW_005202303.1	261237	T	G
**O**	NW_005201183.1	305255	A	G
**O**	NW_005195368.1	310983	A	G
**O**	NW_005198167.1	372840	T	G
**O**	NW_005197128.1	483903	A	G
**O**	NW_005199194.1	530918	T	C
**O**	NW_005200784.1	560813	T	G
**O**	NW_005196756.1	1007040	T	C
**O**	NW_005201392.1	1174477	T	C
**O**	NW_005196756.1	1397008	C	G
**O**	NW_005197847.1	21107	A	C
**O**	NW_005195300.1	80357	A	G
**O**	NW_005199526.1	1566	T	C
**O**	NW_005198243.1	129818	T	G
**O**	NW_005195584.1	475932	T	G
**R**	NW_005200861.1	9261	C	G
**R**	NW_005196755.1	395413	G	A
**R**	NW_005200600.1	480852	T	C
**R**	NW_005196830.1	507541	C	G
**R**	NW_005200690.1	953742	G	C
**R**	NW_005199803.1	3106	C	A
**R**	NW_005198966.1	2267119	G	A
**R**	NW_005200417.1	320924	C	T
**R**	NW_005200982.1	900682	G	A
**R**	NW_005201719.1	6190	T	C
**R**	NW_005201231.1	48878	G	A
**R**	NW_005199478.1	100669	T	C
**R**	NW_005200333.1	43224	G	C
**R**	NW_005200250.1	852053	C	G
**R**	NW_005199770.1	1889462	C	G
**R**	NW_005201213.1	94192	G	C
**R**	NW_005201412.1	223023	T	C
**R**	NW_005200690.1	985680	T	G
**R**	NW_005198785.1	45101	C	A
**R**	NW_005196310.1	383495	C	G
**R**	NW_005199188.1	47672	C	T
**S**	NW_005196295.1	1964	C	T
**S**	NW_005197034.1	468	G	T

**E**	NW_005199194.1	765536	C	T		**S**	NW_005197984.1	625	C	T
**E**	NW_005200690.1	868103	T	C		**S**	NW_005199962.1	4100	G	T
**E**	NW_005198751.1	177019	A	C		**S**	NW_005197465.1	16440	C	G
**E**	NW_005201792.1	1213871	A	G		**S**	NW_005197862.1	20233	G	A
**E**	NW_005200250.1	1026751	A	T		**S**	NW_005199298.1	38740	T	G
**E**	NW_005200892.1	10359	C	G		**S**	NW_005199333.1	38975	C	A
**E**	NW_005201113.1	109212	T	A		**S**	NW_005198644.1	49347	G	A
**G**	NW_005199250.1	5055	A	C		**S**	NW_005200982.1	55312	C	T
**G**	NW_005201608.1	6016	G	A		**S**	NW_005198801.1	62281	C	T
**G**	NW_005200941.1	14889	C	A		**S**	NW_005199553.1	62894	T	C
**G**	NW_005199821.1	28047	G	A		**S**	NW_005202113.1	72930	T	C
**G**	NW_005199434.1	31319	T	C		**S**	NW_005198714.1	81376	C	T
**G**	NW_005200670.1	63696	G	A		**S**	NW_005199796.1	82561	A	G
**G**	NW_005200218.1	76831	G	A		**S**	NW_005199968.1	121813	A	T
**G**	NW_005199925.1	96083	G	T		**S**	NW_005201551.1	127070	G	A
**G**	NW_005199530.1	101734	A	G		**S**	NW_005200803.1	135013	C	G
**G**	NW_005200138.1	115505	C	A		**S**	NW_005198243.1	151154	T	C
**G**	NW_005200871.1	133527	C	T		**S**	NW_005200250.1	154124	T	C
**G**	NW_005196755.1	154311	A	T		**S**	NW_005201613.1	182256	C	T
**G**	NW_005196094.1	205232	G	T		**S**	NW_005200562.1	182599	C	G
**G**	NW_005201249.1	239109	G	T		**S**	NW_005200861.1	235731	C	T
**G**	NW_005197351.1	241737	C	A		**S**	NW_005195085.1	269691	G	A
**G**	NW_005201113.1	250031	T	C		**S**	NW_005199770.1	276468	T	A
**G**	NW_005196755.1	254748	C	G		**S**	NW_005201223.1	310489	G	A
**G**	NW_005201240.1	286968	C	G		**S**	NW_005200881.1	331678	T	C
**G**	NW_005201047.1	296580	C	T		**S**	NW_005199194.1	343330	C	G
**G**	NW_005195368.1	297037	A	G		**S**	NW_005198467.1	455110	G	A
**G**	NW_005195300.1	299330	C	T		**S**	NW_005201092.1	460240	A	G
**G**	NW_005196456.1	323612	G	A		**S**	NW_005198391.1	505118	A	G
**G**	NW_005201360.1	336647	G	A		**S**	NW_005201287.1	547308	G	T
**G**	NW_005198611.1	344667	T	C		**S**	NW_005201792.1	633874	C	G
**G**	NW_005198966.1	354759	G	C		**S**	NW_005200982.1	673744	C	T
**G**	NW_005199045.1	394884	T	C		**S**	NW_005198966.1	922092	G	A
**G**	NW_005201412.1	417940	G	A		**S**	NW_005200690.1	993140	C	T
**G**	NW_005196163.1	479279	A	G		**S**	NW_005202428.1	1005329	A	G
**G**	NW_005198167.1	563621	C	T		**S**	NW_005195301.1	1011699	G	A
**G**	NW_005196021.1	593645	A	T		**S**	NW_005199770.1	2289920	A	G
**G**	NW_005201092.1	600292	C	A		**S**	NW_005201014.1	268611	A	G
**G**	NW_005198966.1	775474	T	A		**S**	NW_005197877.1	102680	A	G
**G**	NW_005199363.1	814637	C	T		**S**	NW_005195301.1	290394	A	G
**G**	NW_005200507.1	817216	T	C		**S**	NW_005200911.1	294631	A	G
**G**	NW_005200600.1	919348	G	A		**S**	NW_005201192.1	572617	A	G
**G**	NW_005196756.1	1033000	A	G		**S**	NW_005199836.1	8464	C	A
**G**	NW_005198244.1	1612203	T	A		**S**	NW_005199045.1	660845	G	C
**G**	NW_005202428.1	2217863	C	A		**S**	NW_005200835.1	71063	T	C
**G**	NW_005200417.1	268700	A	C		**S**	NW_005196830.1	155349	T	C
**G**	NW_005197664.1	14229	T	G		**S**	NW_005200722.1	171117	T	C
**H**	NW_005199111.1	1391	G	T		**S**	NW_005201381.1	364031	T	C
**H**	NW_005200461.1	21887	T	G		**S**	NW_005198966.1	1316153	T	C
**H**	NW_005200793.1	26911	A	G		**S**	NW_005200250.1	792057	T	G
**H**	NW_005199591.1	84265	A	G		**T**	NW_005195227.1	652	G	T

**H**	NW_005197198.1	94802	C	T
**H**	NW_005196756.1	119710	G	T
**H**	NW_005202303.1	133484	G	A
**H**	NW_005196900.1	140610	C	G
**H**	NW_005197198.1	186721	A	C
**H**	NW_005201014.1	187519	C	G
**H**	NW_005198244.1	239517	T	C
**H**	NW_005199926.1	266973	T	A
**H**	NW_005198017.1	274524	G	A
**H**	NW_005198751.1	288177	T	C
**H**	NW_005195300.1	317236	C	T
**H**	NW_005196020.1	328587	T	C
**H**	NW_005197576.1	438552	G	C
**H**	NW_005200011.1	480905	C	G
**H**	NW_005198900.1	530421	G	T
**H**	NW_005198391.1	592065	C	A
**H**	NW_005200011.1	609126	C	A
**H**	NW_005200417.1	887263	T	C
**H**	NW_005201792.1	970994	A	G
**H**	NW_005201287.1	980816	C	G
**H**	NW_005196756.1	1251608	T	C
**H**	NW_005202428.1	2229143	G	C
**H**	NW_005202428.1	2969723	G	A
**H**	NW_005198167.1	460787	C	G
**H**	NW_005198768.1	39712	A	G
**H**	NW_005199861.1	104541	A	C
**H**	NW_005198900.1	243352	A	G
**H**	NW_005195301.1	1151367	A	G
**H**	NW_005195368.1	123589	C	G
**H**	NW_005200881.1	744753	C	G
**H**	NW_005200517.1	152430	G	A
**H**	NW_005199981.1	1333	T	A
**H**	NW_005200730.1	90596	T	A
**H**	NW_005198016.1	16698	T	C
**H**	NW_005201421.1	118456	T	C
**H**	NW_005199770.1	1532038	T	C
**H**	NW_005198380.1	5646	T	G
**H**	NW_005198729.1	27891	T	G
**H**	NW_005201080.1	32333	T	G
**H**	NW_005196528.1	179572	T	G
**J**	NW_005201187.1	1238	A	G
**J**	NW_005201612.1	6936	A	G
**J**	NW_005195641.1	8757	T	A
**J**	NW_005196379.1	10609	T	C
**J**	NW_005196230.1	12266	C	T
**J**	NW_005197273.1	20954	G	C
**J**	NW_005201113.1	27043	C	T
**J**	NW_005200793.1	29526	G	T
**J**	NW_005198001.1	31070	G	T
**J**	NW_005200993.1	35240	A	G
**J**	NW_005201269.1	51255	A	C
**T**	NW_005195271.1	912	C	A
**T**	NW_005197643.1	1003	A	G
**T**	NW_005197947.1	870	C	T
**T**	NW_005198867.1	510	A	T
**T**	NW_005200624.1	569	A	G
**T**	NW_005200812.1	4316	T	C
**T**	NW_005199171.1	8398	G	A
**T**	NW_005199925.1	11522	G	C
**T**	NW_005198921.1	20590	T	G
**T**	NW_005199016.1	25650	C	T
**T**	NW_005197678.1	27782	G	T
**T**	NW_005201269.1	29219	C	A
**T**	NW_005198296.1	43485	A	G
**T**	NW_005199870.1	45583	T	C
**T**	NW_005200241.1	47709	C	T
**T**	NW_005201912.1	64559	T	C
**T**	NW_005199402.1	68599	A	G
**T**	NW_005195300.1	100659	G	C
**T**	NW_005201144.1	131047	A	C
**T**	NW_005200309.1	135882	T	G
**T**	NW_005200871.1	137917	T	G
**T**	NW_005201338.1	177813	T	C
**T**	NW_005200738.1	185780	T	G
**T**	NW_005200763.1	186653	A	G
**T**	NW_005201102.1	187055	T	C
**T**	NW_005196379.1	189798	T	G
**T**	NW_005200835.1	190220	T	C
**T**	NW_005201493.1	227046	C	T
**T**	NW_005200600.1	243461	A	G
**T**	NW_005200589.1	263734	C	T
**T**	NW_005201327.1	384706	T	A
**T**	NW_005198965.1	488389	T	G
**T**	NW_005196379.1	504730	A	C
**T**	NW_005195736.1	524077	T	G
**T**	NW_005196756.1	589623	G	T
**T**	NW_005196021.1	594568	G	C
**T**	NW_005200784.1	691948	A	G
**T**	NW_005199770.1	863067	A	C
**T**	NW_005200690.1	912406	T	C
**T**	NW_005201192.1	1022506	A	G
**T**	NW_005201092.1	1091982	T	C
**T**	NW_005198461.1	10083	T	C
**T**	NW_005199702.1	74319	T	G
**T**	NW_005198966.1	1940039	A	G
**T**	NW_005199770.1	1481057	T	C
**T**	NW_005200754.1	235893	G	T
**U**	NW_005197198.1	14025	G	A
**U**	NW_005198391.1	35518	A	G
**U**	NW_005198339.1	37348	G	T
**U**	NW_005199953.1	54516	C	A
**U**	NW_005200970.1	74810	G	A

**J**	NW_005196603.1	55550	T	G		**U**	NW_005200993.1	116347	T	C
**J**	NW_005199935.1	59912	G	C		**U**	NW_005200571.1	233374	T	G
**J**	NW_005199314.1	79428	C	G		**U**	NW_005196020.1	387726	A	G
**J**	NW_005200095.1	81169	A	T		**U**	NW_005196756.1	446531	C	G
**J**	NW_005199273.1	94904	T	C		**U**	NW_005196755.1	464635	T	C
**J**	NW_005196528.1	135385	C	T		**U**	NW_005200170.1	533339	A	G
**J**	NW_005199907.1	147534	A	G		**U**	NW_005196456.1	541647	A	G
**J**	NW_005198965.1	229039	G	T		**U**	NW_005200011.1	855506	G	C
**J**	NW_005201102.1	252426	A	G		**U**	NW_005198966.1	925065	C	T
**J**	NW_005198900.1	302288	G	C		**U**	NW_005196756.1	1116602	A	C
**J**	NW_005197648.1	361874	T	C		**U**	NW_005200670.1	166976	T	G
**J**	NW_005199601.1	367169	T	A		**U**	NW_005196528.1	3915	C	G
**J**	NW_005196021.1	405262	C	G		**U**	NW_005199478.1	48483	C	G
**J**	NW_005198611.1	406722	T	C		**U**	NW_005201327.1	286871	G	A
**J**	NW_005198966.1	470396	T	C		**U**	NW_005197648.1	428693	T	G
**J**	NW_005202368.1	505636	C	T		**X**	NW_005195564.1	5632	G	A
**J**	NW_005197128.1	507859	G	C		**X**	NW_005197243.1	7738	C	T
**J**	NW_005198091.1	509484	G	T		**X**	NW_005199975.1	8136	A	T
**J**	NW_005198244.1	522112	G	C		**X**	NW_005198729.1	52350	C	T
**J**	NW_005201092.1	547884	C	G		**X**	NW_005199983.1	54141	C	T
**J**	NW_005200333.1	682639	A	G		**X**	NW_005196830.1	64127	G	A
**J**	NW_005201092.1	706089	A	G		**X**	NW_005199803.1	82716	G	C
**J**	NW_005199770.1	939486	G	A		**X**	NW_005199363.1	156421	C	G
**J**	NW_005201792.1	960957	A	G		**X**	NW_005200738.1	162809	T	A
**J**	NW_005198244.1	995389	A	G		**X**	NW_005198244.1	179873	A	G
**J**	NW_005200784.1	1079864	A	C		**X**	NW_005198017.1	210533	A	G
**J**	NW_005198091.1	253387	T	C		**X**	NW_005198537.1	467271	G	A
**J**	NW_005201191.1	222263	G	T		**X**	NW_005199274.1	476341	T	C
**J**	NW_005201165.1	187235	A	G		**X**	NW_005196456.1	499151	A	T
**M**	NW_005195243.1	677	T	G		**X**	NW_005198167.1	507667	G	T
**M**	NW_005198991.1	749	C	T		**X**	NW_005197648.1	554587	T	C
**M**	NW_005200667.1	1331	A	T		**X**	NW_005199770.1	874697	T	C
**M**	NW_005200871.1	1661	G	C		**X**	NW_005199770.1	956903	T	G
**M**	NW_005197967.1	7188	C	A		**X**	NW_005198966.1	1989648	T	G
**M**	NW_005199626.1	10299	T	C		**X**	NW_005202428.1	2378938	A	G
**M**	NW_005200784.1	30896	T	G		**X**	NW_005195523.1	11124	G	A
**M**	NW_005200552.1	54990	C	G		**X**	NW_005196754.1	2904	A	C
**M**	NW_005199962.1	63844	A	C		**X**	NW_005199870.1	97646	G	T
**M**	NW_005199641.1	126068	A	G		**Y**	NW_005199314.1	7657	C	T
**M**	NW_005200670.1	178811	G	C		**Y**	NW_005199885.1	33905	G	A
**M**	NW_005197576.1	214714	C	A		**Y**	NW_005199289.1	85682	G	A
**M**	NW_005201286.1	373200	T	C		**Y**	NW_005200793.1	86042	T	C
**M**	NW_005198537.1	383619	G	T		**Y**	NW_005200580.1	97178	T	A
**M**	NW_005201318.1	396001	G	A		**Y**	NW_005200011.1	130802	G	A
**M**	NW_005201493.1	404710	T	A		**Y**	NW_005200814.1	183029	T	C
**M**	NW_005197426.1	457948	C	A		**Y**	NW_005196094.1	194879	A	T
**M**	NW_005199601.1	865592	C	G		**Y**	NW_005195301.1	213908	A	G
**M**	NW_005200507.1	886829	G	C		**Y**	NW_005199770.1	229236	C	A
**N**	NW_005202141.1	10369	G	A		**Y**	NW_005201092.1	271405	A	T
**N**	NW_005198785.1	18226	C	T		**Y**	NW_005201202.1	307720	A	G
**N**	NW_005198921.1	21924	G	C		**Y**	NW_005195300.1	316907	G	A

**N**	NW_005196021.1	22347	C	T
**N**	NW_005200378.1	54183	C	T
**N**	NW_005200218.1	77788	A	G
**N**	NW_005201732.1	101504	G	T
**N**	NW_005199577.1	103318	T	C
**N**	NW_005200401.1	110971	C	G
**N**	NW_005200146.1	156204	C	T
**N**	NW_005201360.1	245990	G	A
**N**	NW_005200793.1	256172	T	C
**N**	NW_005199684.1	354151	T	C
**N**	NW_005198611.1	358097	T	A
**N**	NW_005196456.1	436336	T	C
**N**	NW_005196900.1	443088	T	C
**N**	NW_005201287.1	782532	T	G
**N**	NW_005202428.1	1245673	T	G
**N**	NW_005199770.1	1513980	T	C
**N**	NW_005202428.1	1906147	G	A
**N**	NW_005199770.1	2477851	G	A
**N**	NW_005200690.1	24268	T	C
**N**	NW_005197197.1	5660	T	G
**N**	NW_005200880.1	62367	G	T
**N**	NW_005202303.1	451454	A	C
**N**	NW_005196379.1	33895	T	C
**N**	NW_005198360.1	11065	T	G
**N**	NW_005199443.1	191637	T	C
**N**	NW_005201269.1	59480	G	A
**N**	NW_005200784.1	791480	C	G

**Y**	NW_005201287.1	535744	T	G
**Y**	NW_005200250.1	601849	G	C
**Y**	NW_005198244.1	1070685	G	T
**Y**	NW_005196756.1	1431501	C	G
**Y**	NW_005202428.1	1919454	C	T
**Y**	NW_005202237.1	424228	G	T
**Y**	NW_005200385.1	164876	A	G
**Y**	NW_005201421.1	259354	G	C
**Y**	NW_005201316.1	4893	T	C
**Y**	NW_005202237.1	24112	T	C
**Y**	NW_005201249.1	317437	T	C
**Y**	NW_005195441.1	500515	T	G
**Z**	NW_005199635.1	12921	G	A
**Z**	NW_005198444.1	30979	T	G
**Z**	NW_005200543.1	100869	G	A
**Z**	NW_005196021.1	262534	C	A
**Z**	NW_005198900.1	586154	G	A
**Z**	NW_005196756.1	916166	G	A
**Z**	NW_005199944.1	129523	A	C
**Z**	NW_005200803.1	136333	A	G
**Z**	NW_005200088.1	55299	T	C
**Z**	NW_005200478.1	111888	G	C
**Z**	NW_005199722.1	77037	C	A
**Z**	NW_005200623.1	60867	G	T
**N**	Mitochondrion	89	G	A
**U**	Mitochondrion	1605	G	A
**S**	Mitochondrion	6357	T	C
**T**	Mitochondrion	19414	T	C
**T**	Mitochondrion	21640	C	T
**U**	Mitochondrion	25493	T	C
**R**	Mitochondrion	28266	T	C
**U**	Chloroplast	1922	T	C
**G**	Chloroplast	99511	G	A

Table S3. Mutations tested and verified by PCR and Sanger sequencing.

Contig	Position	Ref	Alt	MA_line	Verified?
NW_005199194.1	765536	C	T	E	TRUE
NW_005198017.1	274524	G	A	H	TRUE
NW_005195584.1	355947	T	G	A	TRUE
NW_005200250.1	154124	T	C	S	TRUE
NW_005195301.1	1011699	G	A	S	TRUE

Table S4. Preferred (*) and unpreferred codons in *E. huxleyi* genome.

Aminoacid	Codon	Aminoacid	Codon
Phe	UUU	Pro	CCU
	UUC*		CCC
Leu	UUA		CCA
	UUG		CCG*
	CUU	Thr	ACU
	CUC*		ACC
	CUA		ACA
	CUG*		ACG*
Ile	AUU	Ala	GCU
	AUC*		GCC
	AUA		GCA
Met	AUG		GCG*
Val	GUU	Cys	UGU
	GUC		UGC*
	GUA	Trp	UGG
	GUG*	Arg	CGU
Tyr	UAU		CGC*
	UAC*		CGA
His	CAU		CGG*
	CAC*	Ser	AGU
Gln	CAA		AGC
	CAG*	Arg	AGA
Asn	AAU		AGG*
	AAC*	Gly	GGU
Lys	AAA		GGC*
	AAG*		GGA
Asp	GAU		GGG*
	GAC*	Stop	UAA
Glu	GAA	Stop	UGA
	GAG*	Stop	UAG
Ser	UCU		
	UCC*		
	UCA		
	UCG*		

Note: * means significant preferred codon at 0.01 Pvalue.

Table S5. Sources of data used in the Figure 3.

Species	μ	Ne	Ref
Homo sapiens	1.29E-08	2.00E+04	(Besenbacher et al. 2016)
Mus musculus	5.40E-09	2.00E+05	(Uchimura et al. 2015)
Ficedula albicollis	4.60E-09	4.50E+05	(Smeds et al. 2016)
Arabidopsis thaliana	7.00E-09	2.50E+05	(Ossowski et al. 2010)
Caenorhabditis elegans	1.48E-09	8.00E+04	(Denver et al. 2012)
Caenorhabditis briggsae	1.34E-09	6.00E+04	(Denver et al. 2012)
Drosophila melanogaster	5.49E-09	1.15E+06	(Schrider et al. 2013)
Heliconius melpomene	2.90E-09	2.00E+06	(Keightley et al. 2014)
Daphnia pulex	2.30E-09	8.20E+05	(Flynn et al. 2016)
Pristionchus pacificus	2.00E-09	1.80E+06	(Weller et al. 2014)
Ostreococcus tauri	4.19E-10	2.00E+07	(Blanc-Mathieu et al. 2017)
Chlamydomonas reinhardtii	9.63E-10	3.10E+07	(Ness et al. 2015)
Phaeodactylum tricornutum	4.70E-10	8.70E+06	(Krasovec et al. 2019)
Emiliania huxleyi	5.50E-10	2.70E+06	This study
Saccharomyces cerevisiae	1.67E-10	3.20E+06	(Zhu et al. 2014)
Schizoaccharomyces pombe	2.00E-10	2.60E+06	(Farlow et al. 2015)
Paramecium tetraurelia	1.94E-11	1.24E+08	(Sung, Tucker, et al. 2012)
Tetrahymena thermophila	7.61E-12	1.12E+08	(Long et al. 2016)
Bacillus subtilis	3.28E-10	6.30E+07	(Sung et al. 2015)
Escherichia coli	2.20E-10	1.80E+08	(Lee et al. 2012)
Mesoplasma florum	9.78E-09	1.10E+06	(Sung, Ackerman, et al. 2012)
Pseudomonas aeruginosa	7.92E-11	2.00E+07	(Dettman et al. 2016)
Vibrio cholerae	1.07E-10	1.62E+08	(Dillon et al. 2016)
Vibrio fischeri	2.07E-10	4.78E+08	(Dillon et al. 2016)
Ruegeria pomeroyi	1.39E-10	3.00E+08	(Sun et al. 2017)
Chironomus riparius	2.10E-09	1.40E+06	(Oppold and Pfenninger 2017)
Clupea harengus	2.00E-09	4.00E+05	(Feng et al. 2017)
Pan troglodytes	1.20E-08	2.90E+04	(Venn et al. 2014)
Spirodela polyrhiza	2.38E-10	9.80E+05	(Xu et al. 2019)

NOTE – Mutation rates come from papers listed in the right-hand column in that table below. Effective population size estimates were obtained from the papers cited in the right column or from the Supplementary Table S11 of (Krasovec et al. 2017), the Table 3 of (Feng et al. 2017) and the Supplementary Tables S8 and S10 of (Sung, Ackerman, et al. 2012).

Supplementary references

Besenbacher S, Sulem P, Helgason A, Helgason H, Kristjansson H, Jonasdottir Aslaug, Jonasdottir Adalbjorg, Magnusson OT, Thorsteinsdottir U, Masson G, et al. 2016. Multi-nucleotide de novo mutations in humans. *PLoS Genet*. 12:e1006315.

Blanc-Mathieu R, Krasovec M, Hebrard M, Yau S, Desgranges E, Martin J, Schackwitz W, Kuo A, Salin G, Donnadieu C, et al. 2017. Population genomics of picophytoplankton unveils novel chromosome hypervariability. *Science Advances* 3:e1700239.

Denver DR, Wilhelm LJ, Howe DK, Gafner K, Dolan PC, Baer CF. 2012. Variation in base-substitution mutation in experimental and natural lineages of *Caenorhabditis nematodes*. *Genome Biol Evol*. 4:513–522.

Dettman JR, Sztepanacz JL, Kassen R. 2016. The properties of spontaneous mutations in the opportunistic pathogen *Pseudomonas aeruginosa*. *BMC Genomics* 17:27.

Dillon MM, Sung W, Sebra R, Lynch M, Cooper VS. 2016. Genome-wide biases in the rate and molecular spectrum of spontaneous mutations in *Vibrio cholerae* and *Vibrio fischeri*. *Mol Biol Evol*: 34:93-109.

Farlow A, Long H, Arnoux S, Sung W, Doak TG, Nordborg M, Lynch M. 2015. The spontaneous mutation rate in the fission yeast *Schizosaccharomyces pombe*. *Genetics* 201:737-744.

Feng C, Pettersson M, Lamichhaney S, Rubin C-J, Rafati N, Casini M, Folkvord A, Andersson L. 2017. Moderate nucleotide diversity in the Atlantic herring is associated with a low mutation rate. *Elife* 6.

Flynn JM, Chain FJJ, Schoen DJ, Cristescu ME. 2017. Spontaneous mutation accumulation in *Daphnia pulex* in selection-free versus competitive environments. *Mol Biol Evol*. 34:160-173.

Keightley PD, Pinharanda A, Ness RW, Simpson F, Dasmahapatra KK, Mallet J, Davey JW, Jiggins CD. 2014. Estimation of the spontaneous mutation rate in *Heliconius melpomene*. *Mol Biol Evol*. 32:239-243.

Krasovec M, Eyre-Walker A, Sanchez-Ferandin S, Piganeau G. 2017. Spontaneous mutation rate in the smallest photosynthetic eukaryotes. *Mol Biol Evol*. 34:1770–1779.

Krasovec M, Sanchez-Brosseau S, Piganeau G. 2019. First estimation of the spontaneous mutation rate in Diatoms. *Genome Biol Evol.* 11:1829–1837.

Lee H, Popodi E, Tang H, Foster PL. 2012. Rate and molecular spectrum of spontaneous mutations in the bacterium *Escherichia coli* as determined by whole-genome sequencing. *Proc Natl Acad Sci USA* 109:E2774-2783.

Long H, Winter DJ, Chang AY-C, Sung W, Wu SH, Balboa M, Azevedo RBR, Cartwright RA, Lynch M, Zufall RA. 2016. Low base-substitution mutation rate in the germline genome of the ciliate *Tetrahymena thermophila*. *Genome Biol Evol*. 8:3629-3639.

Ness RW, Morgan AD, Vasanthakrishnan RB, Colegrave N, Keightley PD. 2015. Extensive de novo mutation rate variation between individuals and across the genome of *Chlamydomonas reinhardtii*. *Genome Res*. 25:1739–1749.

Oppold A-M, Pfenninger M. 2017. Direct estimation of the spontaneous mutation rate by short-term mutation accumulation lines in *Chironomus riparius*. *Evolution Letters* 1:86–92.

Ossowski S, Schneeberger K, Lucas-Lledó JI, Warthmann N, Clark RM, Shaw RG, Weigel D, Lynch M. 2010. The rate and molecular spectrum of spontaneous mutations in *Arabidopsis thaliana*. *Science* 327:92–94.

Schrider DR, Houle D, Lynch M, Hahn MW. 2013. Rates and genomic consequences of spontaneous mutational events in *Drosophila melanogaster*. *Genetics* 194:937–954.

Smeds L, Qvarnstrom A, Ellegren H. 2016. Direct estimate of the rate of germline mutation in a bird. *Genome Res*. 26:1211-1218.

Sun Y, Powell KE, Sung W, Lynch M, Moran MA, Luo H. 2017. Spontaneous mutations of a model heterotrophic marine bacterium. *ISME J.* 11:1713-1718

Sung W, Ackerman MS, Gout J-F, Miller SF, Williams E, Foster PL, Lynch M. 2015. Asymmetric context-dependent mutation patterns revealed through mutation–accumulation experiments. *Mol Biol Evol*. 32:1672–1683.

Sung W, Ackerman MS, Miller SF, Doak TG, Lynch M. 2012. Drift-barrier hypothesis and mutation-rate evolution. *Proc Natl Acad Sci U S A* 109:18488–18492.

Sung W, Tucker AE, Doak TG, Choi E, Thomas WK, Lynch M. 2012. Extraordinary genome stability in the ciliate *Paramecium tetraurelia*. *Proc Natl Acad Sci U S A*. 109:19339–19344.

Uchimura A, Higuchi M, Minakuchi Y, Ohno M, Toyoda A, Fujiyama A, Miura I, Wakana S, Nishino J, Yagi T. 2015. Germline mutation rates and the long-term phenotypic effects of mutation accumulation in wild-type laboratory mice and mutator mice. *Genome Res*. 25:1125-34

Venn O, Turner I, Mathieson I, de Groot N, Bontrop R, McVean G. 2014. Nonhuman genetics. Strong male bias drives germline mutation in chimpanzees. *Science* 344:1272–1275.

Weller AM, Rödelsperger C, Eberhardt G, Molnar RI, Sommer RJ. 2014. Opposing forces of A/T-biased mutations and G/C-biased gene conversions shape the genome of the nematode *Pristionchus pacificus*. *Genetics* 196:1145–1152.

Xu S, Stapley J, Gablenz S, Boyer J, Appenroth KJ, Sree KS, Gershenzon J, Widmer A, Huber M. 2019. Low genetic variation is associated with low mutation rate in the giant duckweed. *Nature Comm.* 10:1243.

Zhu YO, Siegal ML, Hall DW, Petrov DA. 2014. Precise estimates of mutation rate and spectrum in yeast. *Proc Natl Acad Sci U S A* 111:E2310-2318.
